# Antidiabetic Effect of *Passiflora ligularis* Leaves in High Fat-Diet/Streptozotocin-Induced Diabetic Mice

**DOI:** 10.3390/nu16111669

**Published:** 2024-05-29

**Authors:** Diana P. Rey, Sandra M. Echeverry, Ivonne H. Valderrama, Ingrid A. Rodriguez, Luis F. Ospina, Fatima Regina Mena Barreto Silva, Marcela Aragón

**Affiliations:** 1 Departamento de Farmacia, Universidad Nacional de Colombia, Av. Carrera 30 # 45-03 Edif. 450, Bogotá 111321, Colombia; dpreyp@unal.edu.co (D.P.R.); smecheverryg@unal.edu.co (S.M.E.); ihvalderramap@unal.edu.co (I.H.V.); inarodriguezma@unal.edu.co (I.A.R.); lfospinag@unal.edu.co (L.F.O.); 2 Departamento de Bioquímica, Centro de Ciências Biológicas, Universidade Federal de Santa Catarina, Campus Universitário, Rua João Pio Duarte Silva, 241, Sala G301, Florianópolis 88037-000, SC, Brazil; mena.barreto@ufsc.br

**Keywords:** *Passiflora ligularis*, antidiabetic, flavonoids-O-glucoside, antihyperglycemic, antioxidant, antihyperlipidemic

## Abstract

Type 2 diabetes mellitus (T2DM) is a major global public health concern, prompting the ongoing search for new treatment options. Medicinal plants have emerged as one such alternative. Our objective was to evaluate the antidiabetic effect of an extract from the leaves of *Passiflora ligularis* (*P. ligularis*). For this purpose, T2DM was first induced in mice using a high-fat diet and low doses of streptozotocin. Subsequently, an aqueous extract or an ethanolic extract of *P. ligularis* leaves was administered for 21 days. The following relevant results were found: fasting blood glucose levels were reduced by up to 41%, and by 29% after an oral glucose overload. The homeostasis model assessment of insulin resistance (HOMA-IR) was reduced by 59%. Histopathologically, better preservation of pancreatic tissue was observed. Regarding oxidative stress parameters, there was an increase of up to 48% in superoxide dismutase (SOD), an increase in catalase (CAT) activity by 35% to 80%, and a decrease in lipid peroxidation (MDA) by 35% to 80% in the liver, kidney, or pancreas. Lastly, regarding the lipid profile, triglycerides (TG) were reduced by up to 30%, total cholesterol (TC) by 35%, and low-density lipoproteins (LDL) by up to 32%, while treatments increased high-density lipoproteins (HDL) by up to 35%. With all the above, we can conclude that *P. ligularis* leaves showed antihyperglycemic, hypolipidemic, and antioxidant effects, making this species promising for the treatment of T2DM.

## 1. Introduction

T2DM is considered a public health problem since it affects 6.28% of the world’s population, its prevalence rate is 2.5% per year, and it is increasing at a much faster rate in developed countries; therefore, it is necessary to take prevention and treatment measures urgently [1,2]. T2DM is characterized by defective insulin secretion by pancreatic β cells and tissue resistance to insulin responses, causing hyperglycemia, hyperinsulinemia, increased reactive oxygen species (ROS), oxidative stress, and adipose tissue hypertrophy, among other metabolic changes [3,4].

Due to the previous metabolic changes, the treatment of T2DM requires not only the reduction of blood glucose but also the improvement of the lipid profile and insulin resistance index, among others, so the treatment involves lifestyle changes, oral medications, and in some cases, injectable drugs [5,6]. Despite these therapeutic alternatives, the WHO estimates that 80% of the world’s population uses traditional medicine to treat diseases such as T2DM, and in recent years, studies have increased on plants with potential antidiabetic activity, their main bioactive compounds, and their possible mechanisms of action involved [7,8]. The main bioactive compounds that have shown antidiabetic activity are flavonoids, polyphenols, terpenoids, and alkaloids [9,10].

Although various authors have reported a diversity of medicinal plant species with antidiabetic activity, one of the most extensively studied genus has been Passiflora [11,12,13]. Specifically, our research group has focused its attention on the study of *P. ligularis* and its potential use in the treatment of T2DM [14,15]. Although the antihyperglycemic effect of the aqueous extract and ethanol fraction of *P. ligularis* leaves were demonstrated in normoglycemic animals subjected to an oral glucose tolerance test [16,17], it is important to highlight that the normoglycemic conditions are characterized by a better physiological functionality. Therefore, to demonstrate the antidiabetic effect, it is necessary to conduct tests in an animal model in which diabetes has been induced and mimic the pathogenesis of the disease [18].

Given the importance of not only studying the antidiabetic effect of a medicinal plant but also identifying its bioactive compounds, it should be noted that previously, astragalin and isoquercetin, metabolites of the extract and fraction of *P. ligularis* leaves, were shown to improve glucose homeostasis through insulin secretion in isolated rat pancreatic islets and glucose uptake in the soleus muscle, respectively [19,20]. Therefore, it is interesting to evaluate the antidiabetic effect of the extract and fraction where both compounds are found in greater proportion.

Finally, as mentioned above, given the importance of simulating the pathophysiology and symptomatology of T2DM, there are several animal biomodels that involve genetically modified rodents, but one of the most used biomodels is the induction of obesity and chemical pancreatic damage [21,22]. These models used in the present study combine a high-fat diet with low-dose streptozotocin (HFD/STZ) which mimics the generation of insulin resistance, β-cell dysfunction, the metabolic characteristics, cytokine levels, and oxidative stress similar to those observed in patients with T2DM [23,24].

This research aimed to evaluate the antidiabetic activity of the aqueous extract of *P. ligularis* leaves and its respective ethanol fraction obtained using the XAD2 resin. A chronic model of diabetes induced by HFD/STZ was selected to evaluate this pharmacological activity monitoring the blood glucose levels. Likewise, parameters related to oxidative stress and lipid profile were assessed. A histopathological evaluation of the pancreas was performed too.

## 2. Materials and Methods

### 2.1. Chemicals

Ethanol 99.5% (PanReac AppliChem, Darmstadt, Germany) was employed in the process of obtaining the ethanol fraction of *P. ligularis* extract. Formic acid reagent grade 85% (Carlo Erba, Milan, Italy), Acetonitrile HPLC Grade (Merck Rahway, NJ, USA), and ultrapure water (Milli-Q system Millipore^®^ (Merck, Rahway, NJ, USA) were used for liquid chromatographic analysis. Regarding the analytical standards, isoquercetin (Quercetin-3-glucoside, ≥90%, HPLC), astragalin (Kaempferol 3-glucoside, ≥97.0% HPLC), and chrysin (≥98%, HPLC) were acquired from Sigma-Aldrich^®^ (Sigma Chemical Company, St. Louis, MO, USA).

Streptozotocin (572201), glucose (G7021), carboxymethyl cellulose (CMC) (419273), Lipid Peroxidation (MDA) Assay Kit (MAK085), Catalase Assay Kit (CAT100), and SOD Assay Kit (19160) were purchased from Sigma (St. Louis, MO, USA). An ELISA kit, Monobind (2425-300A), was used to determine the serum insulin levels. Cholesterol MR kit (1118005), HDL-Cholesterol kit (1133010), Triglycerides MR kit, and LDL-Cholesterol kit (1133105) were used to measure lipid markers and were acquired from Linear Chemicals S.L.U. Pentobarbital (Montgat, Spain). (Euthanex^®^) used for animal sacrifice was manufactured by INVET S. A. and purchased from a veterinary establishment.

### 2.2. Preparation of the Extract and Fraction of Passiflora ligularis

The leaves of *P. ligularis* were collected in Anolaima, Cundinamarca-Colombia (Longitude: 74°29.97′ W; Latitude: 4°50.0172′ N; Altitude: 1850 m.a.s.l.) through the sample collection permit granted by the Autoridad Nacional de Licencias Ambientales (ANLA) and the Ministerio de Ambiente y Desarrollo, code 38024, resolution 0699 of 26 April 2018. A voucher copy was deposited in the National Herbarium of Colombia (COL 602878).

Obtaining the extract was carried out as described by Rey et al., 2020 [20]., where the leaves of *P. ligularis* were collected in the municipality of Anolaima in the Department of Cundinamarca, Colombia. After collection, the leaves were dried at room temperature and subsequently pulverized using a knife mill. The aqueous extract was prepared by steeping the plant material in water at 90 °C. To obtain the ethanol fraction, the crude extract was agitated with XAD-2 resin and then washed with ethanol.

### 2.3. Chemical Characterization and Quantification of Flavonoids in the Extract and Ethanolic Fraction of P. ligularis Leaves

The total flavonoid content, isoquercetin, and astragalin were quantified using high-performance liquid chromatography (HPLC) following the methodology previously described [17]. The HPLC was performed on an Agilent 1260 Infinity LC system coupled with a diode array detector (DAD). A Phenomenex-Luna^®^ C18 column (150 × 4.6 mm × 5 μm) was used as the stationary phase at a temperature of 45 °C. Quantification was carried out at wavelengths of 260 nm, 265 nm, and 350 nm for isoquercitrin, astragalin, and total flavonoids, respectively. Chrysin was identified and detected at 267 nm using chrysin ≥ 98%, HPLC grade, as an external standard.

### 2.4. Induction of T2DM and Experimental Design

A total of 30 female Swiss mice between 14 and 16 weeks old, were used in the experiment. Of these, 6 animals (*n* = 6) were randomly selected to constitute the normoglycemic control group throughout the study period, receiving a standard diet. This group was not induced with diabetes nor administered any treatment. The remaining 24 animals were fed a high-fat diet for 8 weeks and then received two low doses of streptozotocin (STZ 40 mg/kg) intraperitoneally, dissolved in citrate buffer (pH 4.5), with a 5-day interval between doses. The animals were provided with a 5% glucose solution overnight to prevent drug-induced hypoglycemia [25]. Three days after administering the second dose of STZ, blood glucose levels (BGL) were measured using Accu-Chek Performa^®^ equipment, collecting blood samples from the lateral tail vein through a small incision. Animals with BGLs above 150 mg/dL were considered for further experimentation. These 24 diabetic mice were then randomly divided into 4 groups (*n* = 6) and received the following by oral gavage for the next 21 days:

Vehicle group: Diabetic control mice receiving the vehicle (0.5% CMC *w*/*v* and 0.5% Tween 80 *w*/*v*).
Metformin group: Diabetic mice receiving metformin (250 mg/kg) as a positive control.
Aqueous extract group: Diabetic mice receiving the aqueous extract of leaves of *P. ligularis* (500 mg/kg).
Ethanolic fraction group: Diabetic mice receiving the ethanolic fraction of *P. ligularis* (250 mg/kg).

It is worth mentioning that during the 21-day treatment period, diabetic mice continued to receive a high-fat diet. Additionally, the normoglycemic group was injected with saline instead of STZ and orally administered the vehicle for 21 days to ensure they experienced the same stress conditions as the diabetic mice.

At the end of the experiment, all the animals were anesthetized with pentobarbital (60 mg/kg) and then sacrificed by cervical decapitation [26]. Once the blood samples were obtained, these were centrifuged (3500 rpm, 10 min) and the serum supernatant was collected. The blood and organ samples were conserved to −80 °C until the lipid profile analysis or serum insulin measurement.

### 2.5. Oral Glucose Tolerance Test

This assay was carried out on the 21st day of administering the treatments, following the methodology previously developed in our research group [27]. Animals were fasted four hours before the test. Oral glucose overload (2000 mg glucose/kg) was administered 30 min after each treatment, and BGLs were measured before and after (30, 60, and 90 min) the overload, using the Accu-Check Performa^®^ (Roche Diagnostics, Basel, Switzerland) equipment.

### 2.6. Insulin Resistance Index (HOMA-IR)

For determining the serum insulin levels, an ELISA kit was used. Insulin resistance (IR) was determined according to the homeostasis model assessment index calculated using Equation (1) [28]:

(1) $$HOMA-IR=\frac{\left(plasma\;insulin\;level\;[\mu U/mL]\times fasting\;plasma\;glucose\;[mg/dL]\right)}{405}$$

### 2.7. Histopathological Examination

After being sacrificed, the pancreas was quickly removed, weighed, and placed in a 10% phosphate-buffered formaldehyde solution in a 1:20 ratio to histopathological studies. According to the standard procedure, the samples were embedded in paraffin, cut into 5 µm thick sections, and stained with hematoxylin and eosin stain according to widely known protocols [29].

### 2.8. Biochemical Parameters

#### 2.8.1. Analysis of Oxidative Stress Parameters

At the end of the bioassay, the organs collected (liver, pancreas, and kidney) were used for the estimation of biochemical parameters of superoxide dismutase (SOD) and catalase (CAT) using commercial kits. A commercial kit was employed to estimate the lipid peroxidation as the MDA (Malondialdehyde) levels in the tissue homogenate.

#### 2.8.2. Serum Lipid Profile

The triglycerides (TG), total cholesterol (TC), high-density lipoprotein (HDL), and low-density lipoprotein (LDL) levels were estimated using diagnostic kits.

### 2.9. Statistical Analysis

**Figure 1:**
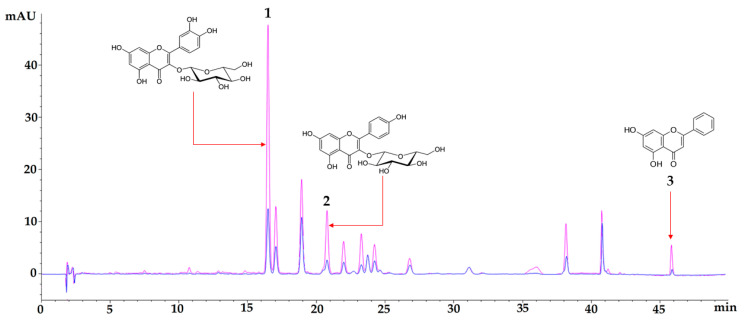
Chromatographic profile of aqueous extract of *Passiflora ligularis* leaves (blue) and an ethanol fraction of *Passiflora ligularis* leaves (pink) at 350 nm. Chromatographic signal 1 corresponds to isoquercetin, signal 2 corresponds to astragalin, and signal 3 corresponds to chrysin. The analytical method used was previously described in the methodology section.

**Figure 2:**
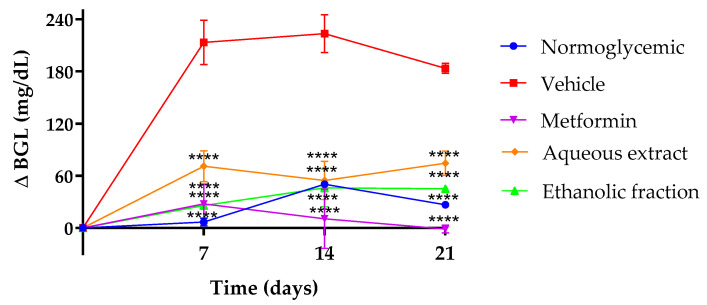
Difference between day 0 and 7, 14, and 21 days in blood glucose levels (BGLs) of the experimental mice. Normoglycemic (blue), vehicle (red), metformin 250 mg/kg (purple), aqueous extract 500 mg/kg (yellow), ethanol fraction 250 mg/kg (green). Data are expressed as mean ± SEM, *n* = 6 animals per group. Two-way ANOVA post-test Bonferroni; **** *p* < 0.0001 with respect to the vehicle group.

**Figure 3:**
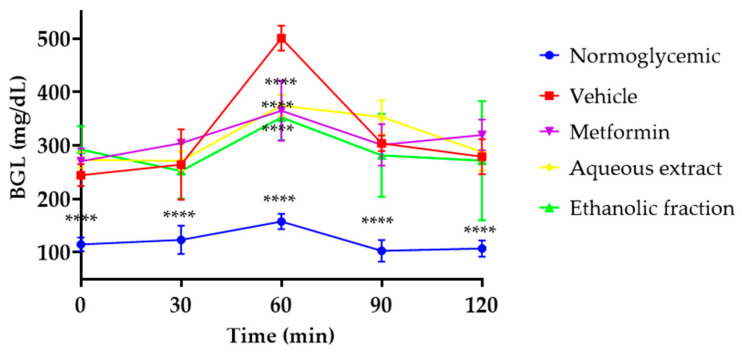
Oral glucose overload test. Normoglycemic (blue), vehicle (red), metformin 250 mg/kg (purple), aqueous extract of *P. ligularis* 500 mg/kg (yellow), ethanol fraction of *P. ligularis* 250 mg/kg (green). Data are expressed as mean ± SEM, *n* = 6 animals per group. Two-way ANOVA post-test Bonferroni; **** *p* < 0.0001 with respect to the vehicle group.

**Figure 4:**
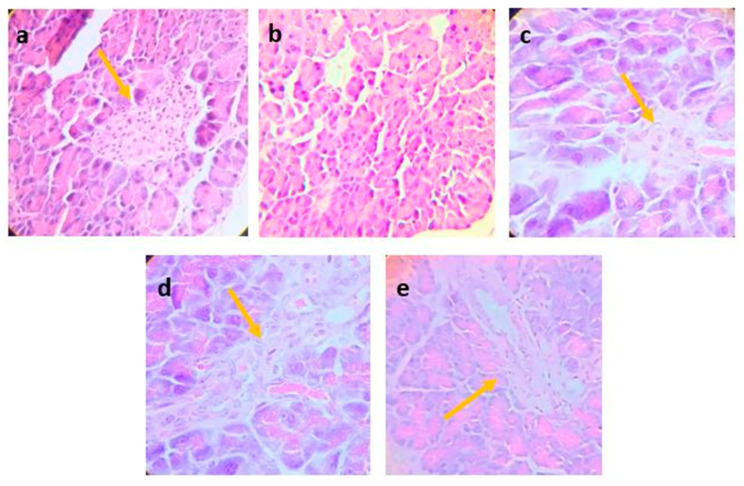
Photomicrographs of pancreatic tissues from different experimental groups were stained by hematoxylin and eosin (H&E) and examined with magnifying power (40×). (**a**) Normoglycemic group, (**b**) Vehicle, (**c**) Metformin, (**d**) Aqueous extract of *P. ligularis*, (**e**) Ethanol fraction of *P. ligularis*. Arrows show the presence of Langerhans islets.

**Figure 5:**
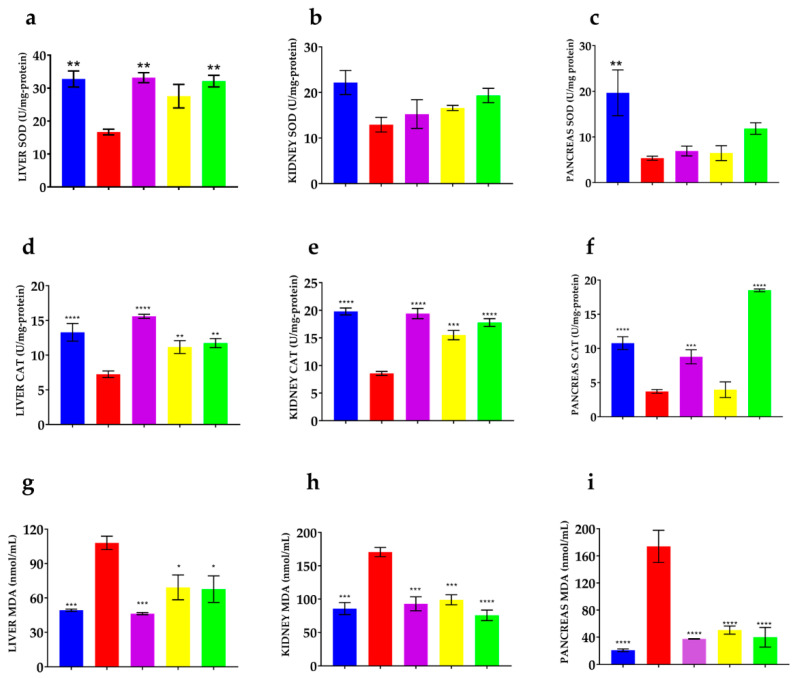
Effect of *P. ligularis* on oxidative stress parameters. Normoglycemic (blue), vehicle (red), metformin 250 mg/kg (purple), aqueous extract of *P. ligularis* 500 mg/kg (yellow), ethanol fraction of *P. ligularis* 250 mg/kg (green). SOD activity: (**a**) liver, (**b**) kidney, (**c**) pancreas; CAT activity: (**d**) liver, (**e**) kidney, (**f**) pancreas; MDA levels: (**g**) liver, (**h**) kidney, (**i**) pancreas. Data are expressed as mean ± SEM, *n* = 6 animals per group. One-way ANOVA post-test Dunnet; * *p* < 0.05, ** *p* < 0.01, *** *p* < 0.001 and **** *p* < 0.0001 with respect to the vehicle group.

GraphPad Prism^®^ software (version 6, San Diego, CA, USA) was employed for data analysis. All results are expressed as mean ± SEM (blood glucose levels, oxidative stress parameters) or mean ± SD (serum lipid profile, fasting glucose, fasting insulin, and HOMA index). We use two-way analysis of variance (ANOVA) followed by Bonferroni post hoc test to analyze the data of Figure 1, Figure 2 and Figure 3, and use a one-way ANOVA followed by post-test Dunnet for the data of Figure 4 and Figure 5, Table 1 and Table 2. Differences were considered significant at *p* ≤ 0.05. The assumptions of ANOVA’s test were evaluated by the software GraphPad prism 6^®^ by the Brown–Forsythe test and Bartlett test for ANOVA.

## 3. Results

### 3.1. Chemical Characterization

Chromatographic analysis was performed for the aqueous extract and ethanol fraction. A concentration of total flavonoids of 60.983 ± 0.755 µg-equivalent of isoquercetin/mg extract was quantified for the aqueous extract, while for the ethanol fraction, the value was 134.998 ± 0.489 µg-equivalent of isoquercetin/mg fraction. As shown in Figure 1, isoquercetin, astragalin, and chrysin in the ethanol fraction showed a significant increase concerning the aqueous extract. The quantified values for isoquercetin and astragalin in the aqueous extract were 12.890 ± 0.376 µg of isoquercetin/mg extract and 4.190 ± 0.042 µg of astragalin/mg extract, respectively. In the ethanol fraction, the content of isoquercetin increased by 241.8% and astragalin by 227.3%, concerning the concentrations found in the aqueous extract of *P. ligularis.*

### 3.2. Effect of P. ligularis on Blood Glucose Levels

Figure 2 presents the fasting blood glucose levels (BGLs) at 7, 14, and 21 days of treatment. As expected, metformin reduced BGLs throughout treatment, the aqueous extract reduced the BGLs by 34%, 27%, and 31% at 7, 14, and 21 days of treatment compared to the vehicle, and the ethanolic fraction reduced BGLs by 36%, 33%, and 41% in the same days in comparison to the vehicle.

### 3.3. Oral Glucose Tolerance Test (OGTT)

In Figure 3, it is possible to see that the normoglycemic mice presented significant differences at all times compared to the vehicle group after oral glucose overload carried out on day 21 after diabetes induction. In contrast, all diabetic mice that received treatment presented with a decrease in BGLs at 60 min. While the vehicle group raised 500.7 ± 23 mg/dL of BGLs, treatments reduced BGLs by 27% for metformin, 25% for the aqueous extract, and 29% for the ethanol fraction of *P. ligularis* with respect to the vehicle group.

### 3.4. Insulin Resistance Index (HOMA-IR)

To measure insulin resistance in HFD/STZ-induced diabetes models, the homeostasis model assessment of insulin resistance (HOMA-IR) is commonly used [30].

According to the results described in Table 1, after 21 days of treatment, fasting glucose decreased by 23% with the aqueous extract and by 32% with the ethanol fraction; meanwhile, fasting insulin decreased by 46% and by 43% for the aqueous extract and ethanol fraction, respectively. The HOMA index decreased by 58% for the extract and 59% for the ethanol fraction; metformin also caused a significant decrease in the three parameters compared to the vehicle group.

### 3.5. Histopathological Examination

According to the photomicrograph shown in Figure 4, it can be observed that diabetic mice, after 21 days of treatment with metformin, aqueous extract, and the ethanol fraction of *P. ligularis*, showed significant preservation of pancreatic islets and exocrine tissue in comparison with the diabetic mice that did not receive treatment. Image A shows normal islets in which the insulin secretion has not been over-stimulated, keeping its structure and size. Micrograph B corresponding to the vehicle group showed cytoplasmic vacuolar degeneration, not the endocrine component. Image C shows a not proliferated islet compared with the other treatments. Image D shows endocrine tissue which is not hypertrophic compared to Figure 4e, which shows a diffused and hypertrophic structure with remarkable preservation of the pancreatic islets compared to the untreated animals. Histological analysis showed that aqueous extract and the ethanol fraction of *P. ligularis* caused a remarkable preservation of pancreatic islets compared to the untreated animals, without an endocrine component.

### 3.6. Analysis of Oxidative Stress Parameters

SOD activity is shown in Figure 5b,c. Significant differences were found between the normoglycemic and vehicle groups in kidney and pancreas tissues. In liver tissue (Figure 5a), the activity of this enzyme increased by 39% for the aqueous extract, 48% for the fraction, and 50% for metformin compared to the vehicle.

Respecting the CAT activity, as shown in Figure 5d, in the liver, the aqueous extract increased the catalase activity by 35%, while the ethanol fraction increased it by 38% compared to the vehicle. According to Figure 4e, in the kidney, the catalase activity increased by 44% in the aqueous extract and 51% for the ethanol fraction compared to the vehicle. Finally, according to Figure 5f, although the aqueous extract did not increase the catalase activity in the pancreas, the ethanol fraction did increase it by 80% with respect to the vehicle. Metformin improved catalase activity in all organs.

Concerning MDA levels, as shown in Figure 5g, the aqueous extract decreased the levels of MDA by 35% and the fraction by 37% compared to the vehicle group. According to Figure 5h, in the kidney, the aqueous extract decreased the levels of MDA by 42% and the fraction by 55% concerning diabetic mice without treatment. Finally, in the pancreas (Figure 5i), the aqueous extract decreased the levels of MDA by 70% and the ethanol fraction by 80% compared to the vehicle. As expected, normoglycemic mice showed higher catalase and SOD activity and lower MDA levels in all organs.

### 3.7. Effect of P. ligularis on Serum Lipid Profile

Due to the relationship between diabetes and hyperlipidemia, the quantification of total triglycerides, high-density lipoprotein cholesterol, low-density lipoprotein cholesterol, and total cholesterol were considered in this study (Table 2).

According to Table 2, TG decreased by 29% for the aqueous extract and by 30% for the ethanol fraction. The total cholesterol parameter decreased by 17% and 35% for the aqueous extract and ethanol fraction, respectively. The LDL-C levels decreased by 24% for the aqueous extract and 32% for the ethanol fraction. Finally, HDL-C increased by 18% for the aqueous extract and 25% for the ethanol fraction compared to the vehicle. For its part, metformin also showed an improvement in the lipid profile.

This effect could be related to the protective effect of the aqueous extract and the ethanol fraction of *P. ligularis* leaves on the pancreas. Figure 4d,e showed that both treatments found endocrine components associated with pancreatic islets. When insulin production or release is deficient, the lipolysis process is not inhibited, causing hyperlipidemia. However, our results showed that the animals treated with the aqueous extract and the ethanol fraction of *P. ligularis* attenuated insulin resistance, which could be related to the decrease in TG, total cholesterol, and LDL-C with the increase in HDL-C compared to diabetic animals administered with the vehicle.

## 4. Discussion

The effect of *P. ligularis* leaves in HFD/STZ-induced diabetic mice was evaluated. The extract and ethanol fraction were assessed for phytochemical identification purposes. According to the chromatographic analysis, isoquercetin, astragalin, and chrysin in the ethanol fraction were found in major amounts compared to the aqueous extract. These findings are in contrast with previous analyses obtained by our research group. It is evident that these flavonoids are found in greater proportion in the fraction, in which, given the nature of the XAD resin, they were expected to be enriched with these polar compounds [31,32].

Other tests were carried out in order to identify changes in diabetic mice after treatment administration. BGL differences were found from day 7. Previously, an extract of *P. glandulosa* fruit showed an antihyperglycemic effect in streptozotocin-induced diabetic mice at 15, 21, and 28 days of treatment, an effect attributed mainly to the presence of flavonoids [33]. The effect of *P. ligularis* on the control of this parameter can also be attributed to flavonoids in the aqueous extract and the ethanol fraction, especially *O*-glycosyl flavonoids such as isoquercetin, astragalin, and chrysin which have been reported previously [15].

With respect to the results obtained in the OGTT test, this test in rodents is a usual test employed to determine if a mouse is glucose intolerant and diabetic. However, this is also useful to determine the hypoglycemic or antihyperglycemic effect of drugs and extracts, among others [34]. Other species of Passiflora have been reported to have a similar antihyperglycemic effect in diabetic models, for example, ethanol extracts of *P. edulis*, methanolic extract of leaves of *P. incarnata*, and *P. foetida* maintain blood glucose levels, improving the oral glucose tolerance in streptozotocin-induced diabetic mice or alloxan diabetic rats [35,36].

In previous studies, the aqueous extract and ethanol fraction of *P. ligularis* leaves demonstrated an antihyperglycemic effect in normoglycemic rats after an oral glucose overload and an increase in glycogenesis [20]. It is noteworthy that this study confirms this effect in diabetic mice after receiving 21 days of treatment considering that the ethanol fraction had a more significant effect. It has been shown that the fraction is rich in flavonoids such as isoquercetin, astragalin, and chrysin [20,37]. This effect may be due to flavonoids since isoquercetin has been shown to decrease BGLs after an oral overload of glucose in diabetic mice associated with an inhibition of the enzyme dipeptidyl peptidase IV (DPP IV) and an increase in serum levels of glucagon-like peptide (GLP-1) and insulin [38].

Another flavonoid that may be involved in the process is astragalin, which has demonstrated a hypoglycemic effect after an oral glucose overload in normoglycemic rats linked to an insulin secretagogue [19]. On the other hand, chrysin also showed an effect of reduction of glucose levels at 60 min after an oral glucose overload in diabetic rats after 30 days of treatment, an effect associated with an increase in the sensitivity of insulin receptors, GLUT-4 glucose transporters, and an increase in muscle glycogen [39].

Given the above, we can suggest that the effects of the aqueous extract and ethanol fraction of *P. ligularis* improve glucose intolerance induced by T2DM and are associated with the presence of the flavonoids mentioned previously.

The homeostasis model assessment of insulin resistance (HOMA-IR) is the most widely used model to determine insulin resistance in rodent models of diabetes since it is a simple, accurate method and causes minimal stress to the animals. This mathematical model relates insulin and fasting plasma glucose with the hyperinsulinemic euglycemic glucose clamp (HEGC) [40]. According to the HOMA-IR results obtained, the extract and the fraction decreased insulin resistance, and this effect had already been evidenced in other passifloras species. For example, the consumption of *P. setacea* juice decreased HOMA-IR in humans, fasting as well as 3 h after consumption [41]. Likewise, the extract of *P. incarnata* leaves prevents insulin resistance, and its effect was strongly associated with the presence of flavonoids [42]. Our results are according to previous reports in the literature since the ethanol fraction decreased HOMA-IR values slightly. In addition, in previous studies, isoquercetin has been shown to decrease HOMA-IR in a dose-dependent manner in type-2-diabetes-induced hepatic injury in rats [43]. Finally, an extract with rich contents of astragalin has also been shown to decrease insulin resistance in a model of diabetes induced with a high-fat diet in rats [44].

According to the previous results, we can suggest that the aqueous extract and especially the ethanolic fraction of *P. ligularis* leaves improved the HOMA index, an effect that can be attributed to the presence of astragalin since it has been shown to increase insulin secretion in the pancreatic tissue by stimulating calcium influx through K^+^_ATP_, L-VDCC channels, and activating protein kinases PKC and PKA [19]. This effect can also be associated with glucose uptake by peripheral tissues such as skeletal muscle, given that isoquercetin, another of the major components, has an insulinomimetic effect by activating the PI3K, MAPK, and MEK/ERK pathways, and de novo protein synthesis of the GLUT-4 transporter [20].

Regarding the histopathological analysis, the disturbance of islet proliferation was observed as result of the diabetes model, which is coherent with previous studies that showed high glucose levels in mice fed with an HFD on islet proliferation but inhibited by metformin administration [45]. Previously, isoquercetin showed protective activity in pancreatic islets showing a similar morphology to normal ones [43]. The results are expected according to the concentration of this molecule within the fraction with respect to the aqueous extract of *P. ligularis.*

Regarding the analysis of the results obtained related to oxidative stress, these parameters were measured since SOD and CAT are antioxidant enzymes that decrease the production of reactive oxygen species (ROS), protect against oxidative stress, nephropathy, and other T2DM complications. CAT also protects pancreatic beta cells from damage caused by hydrogen peroxide (H_2_O_2_). The increase in MDA levels in diabetics suggests that peroxidative lesions are related to the development of diabetic complications and a decrease in antioxidant mechanisms [46].

In this study, only the ethanol fraction was shown to improve the activity of hepatic SOD; however, CAT showed an improvement in all the organs studied, indicating that the antioxidant effect is more related to the metabolism of H_2_O_2_. This effect is evidenced with a higher proportion at the pancreatic level comparable to the normoglycemic group, which confirms the results obtained at the histopathological level. Likewise, since the ethanol fraction had a more significant antioxidant effect, it can be associated with flavonoids such as chrysin, which may be responsible for this effect since it has previously shown a decrease in MDA levels and an increase in SOD and CAT activity in the liver, brain, and pancreas of diabetic rats after 4 weeks of treatment [47]. In the same way, the aqueous extract of *M. oleifera* leaves rich in flavonoids such as isoquercetin and astragalin has shown an increase in the levels of CAT and SOD, and decreased to reduce MDA levels at the liver and kidney level in diabetic rats [48]. Also, an extract of *P. incarnata* extract with flavonoids such as quercetin and kaempferol showed after 30 days of treatment a decrease in the levels of MDA at the liver level in mice that received a high-fat diet [42].

The above information leads us to conclude that *P. ligularis* treatments ameliorate oxidative stress in tissues such as the pancreas, liver, and kidney, which in turn can prevent the onset of complications such as nephropathy, which, as mentioned above, is related to ROS. This effect can be attributed to the presence of isoquercetin or astragalin, although their molecular mechanisms must be studied in depth in future research.

In relation to the results obtained in the lipid profile, is well known that defects in insulin action or hyperglycemia could be associated with changes in plasma lipid/lipoprotein metabolism in patients with diabetes [49]. Hypertriglyceridemia and reduced plasma high-density lipoprotein cholesterol are the most common lipid abnormalities in T2DM [50].

Previous reports have shown that different species of *Passiflora* generate changes in the serum lipid profile of rodents. *Passiflora incarnata* L. decreased the impact of a high-fat diet in insulin-resistant mice, reducing total cholesterol and triglycerides levels and increasing the concentration of high-density lipoproteins (HDL) [42]. Similar behavior was also reported for different extracts of *P. edulis* in diabetic rats, attributed to glycosyl flavonoids [51,52]. Improvements in the lipid profile have been demonstrated by *P. ligularis* in obese rats and is correlated with the decrease in hepatic steatosis [53]. The results of the present study are according to previous studies which demonstrated the antilipidemic activity of isoquercetin in a dependent dosage which attenuated triglycerides and cholesterol levels in diabetic mice [38]. Additionally, an extract with rich contents of astragalin has been shown to decrease cholesterol, triglycerides, LDL, and increase HDL in high-fat-induced and fructose-diet-induced diabetes in Wistar rats [43].

## 5. Conclusions

The results of this study suggest that the aqueous extract and the ethanolic fraction of *P. ligularis* leaves are promising as a complementary or adjuvant treatment for T2DM, since they are not only limited to controlling glucose levels in streptozotocin-induced diabetic mice, but also improve oxidative stress parameters in organs such as the kidney, pancreas, or liver, as well as improve the lipid profile by reducing levels of TG, TC, LDL-C, and increasing levels of HDL-C. Likewise, the oxidative stress parameters were improved, increasing the enzyme activity of SOD and CAT and decreasing the MDA values. It is important to highlight that this would be the first time that an extract from *P. ligularis* leaves has been determined to have a positive effect on several relevant outcomes in the control of T2DM. It is also important to keep in mind that the leaves of this species are generally discarded after the fruit has been grown, so they would also become a usable resource. For subsequent studies, it is recommended to design a formulation through which to administer the extract and carry out studies in humans.

## Figures and Tables

**Table 1:** Effect of the aqueous extract and ethanol fraction of *P. ligularis* on fasting glucose, fasting insulin, and HOMA index.

TREATMENT	Fasting Glucose mg/dL	Insulin Levels (µUI/mL)	HOMA-IR
Normoglycemic	105.600 ± 7.579 ****	2.267 ± 0.332 ****	0.591 ± 0.086 ****
vehicle	434.000 ± 26.069	23.178 ± 2.527	24.830 ± 2.421
Metformin 250 mg/kg	347.286 ± 19.499 ****	15.877 ± 1.752 ****	13.610 ± 1.343 ****
Aqueous extract 500 mg/kg	333.800 ± 15.766 ****	12.493 ± 1.407 ****	10.294 ± 1.037 ****
Ethanol fraction 250 mg/kg	292.600 ± 27.207 ****	12.986 ± 1.217 ****	9.379 ± 0.786 ****

Data are expressed as mean ± SD. *n* = 6 animals per group. One-way ANOVA post-test Dunnet; **** *p* < 0.0001 with respect to the vehicle group.

**Table 2:** Effect of aqueous extract *P. ligularis* 500 mg/kg and ethanol fraction *P. ligularis* on serum lipid profile in diabetic mice.

TREATMENT	Triglyceride (TG) mg/dL	Total Cholesterol mg/dL	LDL-C mg/dL	HDL-C mg/dL
Normoglycemic	96.870 ± 2.230	140.280 ± 15.345	88.820 ± 25.778	37.843 ± 1.926
vehicle	225.506 ± 13.345	252.076 ± 5.479	174.658 ± 17.526	28.923 ± 0.081
Metformin 250 mg/kg	169.606 ± 3.615 ****	159.775 ± 4.805 ****	98.438 ± 13.633 ***	36.727 ± 4.824 **
Aqueous extract 500 mg/kg	159.450 ± 7.070 ****	207.410 ± 17.580 *	131.464 ± 25.953 *	34.263 ± 2.206 *
Extract fraction 250 mg/kg	157.160 ± 0.430 ****	162.700 ± 13.790 ****	117.336 ± 2.449 **	36.237 ± 1.980 **

LDL-C—low-density lipoprotein cholesterol, HDL-C—high-density lipoprotein cholesterol. Data are expressed as mean ± SD. *n* = 6 animals per group. One-way ANOVA post-test Dunnet; * *p* < 0.05, ** *p* < 0.01, *** *p* < 0.001 and **** *p* < 0.0001 with respect to the vehicle group.

## Data Availability

The original contributions presented in the study are included in the article, further inquiries can be directed to the corresponding author.
